# Perspectives on Emotional Care: A Qualitative Study with Cancer Patients, Carers, and Health Professionals

**DOI:** 10.3390/healthcare11040452

**Published:** 2023-02-04

**Authors:** Meinir Krishnasamy, Heidi Hassan, Carol Jewell, Irene Moravski, Tennille Lewin

**Affiliations:** 1 Academic Nursing Unit, Peter MacCallum Cancer Centre, Melbourne, VIC 3000, Australia; 2 Department of Nursing, School of Health Sciences, University of Melbourne, Melbourne, VIC 3052, Australia; 3 Victorian Comprehensive Cancer Centre Alliance, Melbourne, VIC 3000, Australia; 4 Sir Peter MacCallum Department of Oncology, University of Melbourne, Melbourne, VIC 3052, Australia; 5 Department of Epidemiology and Preventive Medicine, Monash University, Melbourne, VIC 3168, Australia

**Keywords:** cancer care, emotional care, person-centered care, qualitative research

## Abstract

The emotional consequences of a cancer diagnosis are well documented and range from emotional distress, defined as suffering associated with feelings such as shock, fear, and uncertainty, through to psychological distress that may manifest as depression, anxiety, feelings of hopelessness, or heightened risk of suicide. This study set out to explore the assumption that the provision of emotional care should be the platform upon which all other aspects of cancer care are delivered and, that without attention to emotional care, no other aspects of cancer care can be fully realized. Utilizing qualitative focus groups and in-depth interviews with 47 patients, carers, and health professionals, emotional care was shown to be (1) fundamental to the provision of comprehensive cancer care, (2) essential to easing the burden of a cancer diagnosis and demands of treatment, (3) everyone’s business, and (4) a component of cancer care at any time and every time. Future studies are needed to test interventions to enhance provision of intentional, purposeful, and individualized emotional care to help patents achieve the best health outcomes possible.

## 1. Introduction

Cancer and its treatments can affect every aspect of an individual’s life, giving rise to a range of supportive care needs that can include informational, physical, practical, social, spiritual, psychological, and emotional requirements [1]. When left unaddressed, these needs can impact capacity to tolerate or adhere to treatment, capacity to engage in treatment decision-making, patient experience and health outcomes, and health system costs [2,3]. Supportive care refers to the provision of services, resources, and interventions required by people to cope with the needs triggered or exacerbated by a diagnosis of cancer, the demands of treatment, and ongoing consequences of a cancer diagnosis [1] and is recognized as a component of quality cancer care [4]. This paper reports on experiences of emotional care as a distinct component of supportive care, as reported by people affected by cancer and health care professionals involved in their care. The data were collected as part of a larger study (described below). The manuscript is not concerned with provision of psychological care for patients who present with or develop complex mental health issues during a cancer experience, which require specialist psychological or psychiatric intervention.

### Cancer, Supportive Care, and Emotional Health

The emotional health consequences of a cancer diagnosis have been well documented and range from emotional distress (suffering associated with feelings such as shock, fear, and uncertainty), through to psychological distress that may manifest as depression, anxiety, feelings of hopelessness, or heightened risk of suicide [5,6]. Approximately 35–40% of patients with cancer experience emotional or psychological distress at some stage during their illness [7,8], and this is especially so for patients who enter the health system already burdened by poverty, poor health literacy, rurality, cultural and linguistic diversity, or who belong to Indigenous or First Nations peoples [9]. Emotional care refers to the identification of and tailored responses to the emotional suffering experienced by people affected by cancer [1].

Furthermore, people affected by some cancers, such as head and neck cancer, are estimated to have suicide rates up to four times higher compared with the general population [10]. Cancer caregivers also experience considerable unmet emotional need, especially when the trajectory of care is prolonged [11] or where prognosis is poor at time at diagnosis [12]. Indeed, several studies have demonstrated that caregivers experience more emotional challenges than patients themselves [13,14]. Combined, patients with advanced cancers and their carers report emotional and psychological needs as the most prevalent unmet supportive care domains [12].

Although timely, targeted, and personalized supportive care screening, assessment, and intervention has been shown to relieve emotional and psychological needs following a cancer diagnosis [15,16,17], provision of supportive care (and thus attention to emotional and psychological needs) remains inconsistent [18]. This is concerning given evidence demonstrates that the emotional wellbeing of patients is linked to their ability to communicate with members of their health care team, make decisions, adhere to treatment, and achieve optimal physical health outcomes [19]. One explanation for this may be the conceptualization of emotional care (as a component of supportive care) as something adjunct or additive to the treatment of cancer care [20], rather than recognizing it as the platform upon which all other aspects of cancer care should be constructed or delivered [4].

The data reported in this paper are drawn from a large mixed-methods study undertaken to refresh and develop new approaches to the integration of cancer supportive care as a component of routine cancer service delivery. The study was funded by the Victorian Department of Health in Australia and approved by the University of Melbourne Human Research Ethics Committee (ID REF: 185 1227.5). A broad community of 300 cancer consumers (patients/family members/support persons), health professionals, health services researchers, policy makers, and not for profit organization members (other participants) took part in the mixed methods study, and data gathered were used to build an online supportive care portal (https://wecan.org.au (accessed on 20 December 2022).) (publication in preparation). Here, we present insights gained from qualitative focus groups and in-depth interviews undertaken as part of the larger study between January–November 2018 regarding provision of emotional care. This paper sets out to report participants’ accounts of experiencing or responding to emotional distress, as defined above. It also sets out to consider whether data generated support the assumption that the provision of emotional care should be the platform upon which all other aspects of cancer care are delivered and, that without attention to emotional care, no other aspects of cancer care can be fully realized.

## 2. Materials and Methods

An exploratory qualitative approach, utilizing data collected during Town Hall and Community of Practice events as part of the larger mixed methods study. Town Hall meetings are a recognized approach to community engagement when a broad and inclusive approach to data collection is required to meet study aims [21]. A Community of Practice is a group of people who come together over a period of time to share an interest in, address, and learn more about a particular topic [22].

### 2.1. Methods

Focus groups were chosen to collect data during the larger supportive care study, to facilitate interaction among a large and diverse group of participants, in order to generate a wide range of views and ideas [23]. Participants who were unable to attend a focus group were offered the opportunity to take part in an in-depth interview.

### 2.2. Recruitment and Consent

Recruitment involved purposive and snowball sampling techniques [24]. Initially, a list of key stakeholders (described above as part of the larger mixed methods study) was developed by the project lead who had experience of and expertise in cancer supportive care (MK). The aim was to purposively recruit a diverse sample of participants with experience of supportive care either as a cancer patient, carer (i.e., family member or informal support person), or health professional. Potential participants were approached directly via email describing the intent of the study and inviting them to take part. The email contained a flyer explaining eligibility criteria, what participation would involve, and details of how to contact a member of the project team to express interest in taking part. Potential participants were asked to forward on the flyer to colleagues/friends they believed met the eligibility criteria and who might be interested in taking part. Eligibility criteria for people affected by cancer were: (1) current or previous cancer diagnosis or family member/support person of somebody who has/had a cancer diagnosis, (2) 18 years of age or over, (3) able to provide informed consent, and (4) able to read, write and speak English or take part with support of a family member/support person. Eligibility criteria for the health professionals were: (1) experience of working with/supporting people affected by cancer in any aspect of the health sector, (2) 18 years of age or over, and (3) able to provide informed consent. Following contact with a member of the project team (MK, IM, CJ), a copy of the Participant Information and Consent Form (PICF) was emailed or mailed to participants to read and sign and bring it along with them on the day of the focus group or interview. Participants who took part in an interview remotely (that is via telephone) were asked to return the signed consent form via email or mail, as preferred. Consent was also re-checked verbally and audio-recorded on the day of the focus group or interview.

### 2.3. Data Collection

After re-checking consent at the beginning of each focus group or interview, participants were asked to complete a brief demographic data collection form to indicate their status as a patient, carer, or health professional, their age, place of residence or work, language spoken at home (patients and carers) or, their professional affiliation (health professionals only). Intentionally, no data on cancer diagnosis, time since treatment completion, or treatments received were collected from patients or carers as there was no intention to undertake any tests of association or to consider experiences of emotional care in the context of a specific diagnosis. As such, collecting these data seemed intrusive. To address any potential imbalance of power by having health professionals, patients, and carers in the same focus groups [25], three patient and carer, and three health professional focus groups were conducted separately. Of the six focus groups, two were held in a regional setting, ensuring that the study was accessible to people affected by cancer and health professionals outside of metropolitan sites. Patient and carer participants were compensated for their travel and parking cost. Focus groups were moderated by two members of the project team (MK, CJ, IM), one to lead discussion and a second to observe and take notes of context and environment and to ensure that all participants had an opportunity to contribute. Interviews were conducted by the same project team members. A semi-structured discussion guide (see Supplemental Material S1) was developed based on data generated by Town Hall meetings and Community of Practice events undertaken as part of the larger mixed methods study. The discussion guide facilitated exploration of participants’ experiences and understanding of supportive care and their views of its value and importance. The questions did not specifically direct participants to emotional domains of supportive care and so the findings reported below represent spontaneous insights recounted by participants of their experiences and views regarding emotional care. An example of questions included in the focus groups and in-depth interviews is presented in Table 1. A full list of focus group and interview questions are available as Supplementary Material S1.

Interviews and focus groups were audio recorded and transcribed verbatim as word documents. Word documents were uploaded to NVivo 12 [26] to help organize and manage the analytical process. All study data were stored in secure electronic folders and only named members of the project team had access to audio-recordings, participants demographic data, and consent forms. Transcribed audio recordings were de-identified.

### 2.4. Analysis

Data analysis was undertaken in accordance with Braun and Clarke’s six steps of thematic analysis [27]. Recordings from each data set were initially listened through, transcripts read, and notes made about general observations and impressions, as part of researcher familiarization and rich engagement with the data. Transcripts were coded by two independent coders (CJ, SM) and any disagreement clarified through discussion with the project lead (MK). A threshold for saturation was agreed prior to data analysis which was agreed as the point at which no new themes were generated through the analytical process. The focus was on producing semantic, rather than latent themes as a means of establishing a detailed description of the data, but without engaging in broader interpretative theorizing [28]. All data were coded line by line using an inductive process. Candidate themes were identified by reviewing the codes and individual codes were collated under candidate themes. Themes were refined and revised using a reflexive process, and consistent with Braun and Clarke’s model, themes were conceptualized as constructs rather than representative of “reality” [28]. Participants’ demographic data were analyzed descriptively. The manuscript has been prepared according to the Consolidated Criteria for Reporting Qualitative Research Checklist [29].

## 3. Results

### 3.1. Participants

Forty-seven participants (16 patients, six carers, and 25 health professionals) took part across six focus groups and eight interviews (Table 2).

### 3.2. Focus Groups/Interviews

Four themes were generated that related to emotional care. No themes were unique to either focus group or interview participants. Themes are illustrated below by inclusion of participant quotes. Where more than one quote is included per theme, is it to illustrate nuance within the theme, rather than being indicative of a theme’s prevalence within the data.

### 3.3. Emotional Care—A Fundamental Component of Cancer Care

People affected by cancer spoke of the provision of emotional care as being necessary to enable a person to live well, to cope throughout the treatment period, and beyond.

*“So there’s emotional care, there’s physical care, there’s managing symptoms of cancer and of treatment. The emotional support of the patient [is important]… so they can live well throughout that time.”*

(P042)

Attending to a person’s emotional needs was recognized by health professionals as fundamental to quality of life and ability to cope with the treatment “journey” and making it the best it could be.

*“what … might be of benefit to that person, wherever they are in their treatment phase… what services … might be of benefit for them to improve quality of life, make their treatment better, make their journey better.”*

(W FG HP01)

People affected by cancer spoke about their experience of emotional care in the context of help, advice, communication, and perhaps most significantly, listening and being responsive. Participants’ emotional wellbeing was described as coming from feeling cared about, having a connection with a health professional who understood their struggles and circumstances, and took time to listen to them.

*“The most important and valuable thing is I guess, yeah, it’s that you’ve got someone to talk to about your emotional state. It’s really about the emotional state I guess. It’s having someone you can talk to about it, … your emotional feelings, just dealing with your anxieties and your emotions really.”*

(PC046)

For some, this was a connection with a particular health professional, often a cancer nurse, while for others, this came through connections made in peer support groups and community services.

*“it can be the simple things like, can I do the shopping or can I come and wash your dishes, and people don’t know what to ask or how to ask if they could help.”*

(M FG HP05)

For many health professionals ensuring access to practical support, delivered by nurses, occupational therapists, dietician, physiotherapists, psychologists, or social workers was regarded as an important emotional intervention where recognizing what a person needed practically, was a way of acknowledging an individual’s unique circumstances, and a way of *‘being with’* a person.

*“For me it means it’s other people to help so that the patient’s got lots of experts to help them with all of the needs that they’ve got.”*

(M FG HP01)

### 3.4. Easing the Burden

For many participants, the provision of emotional care was synonymous with easing the burden of a cancer diagnosis. Easing the burden came in very many forms, from ensuring that personal needs were attended to whilst in hospital, recognizing that someone is experiencing hardship and making it possible to ask for help, finding practical solutions to problems such as living arrangements, getting the shopping, knowing how to tell family about the diagnosis, and recognizing the mounting costs and financial concerns. These were all recognized as the provision of emotional care because they took account of the wider context of the person’s life and the extensive impacts of a cancer diagnosis.

*“[health professionals] need to not just look at us as a physical thing, they need to look at us as the whole person, they need to look at us how we are emotionally and especially in our situations outside, our family situations and work with that as well because that’s what makes us get better and it’s what makes us feel supported if they, you know, that they’re involved and understand what’s going on…”*

(M FG CC 07)

People affected by cancer described being unclear about what support or help was on offer to them, especially soon after diagnosis, and there was a concern that people might miss out on important care as a consequence.

*“if you say to a person do you want [support] or supportive care and they say no, what do they understand that they were gonna get, or what have they just missed out on.”*

(PC041)

Health professionals spoke about the importance of being explicit about what was on offer, of asking gently probing questions to ensure that patients’ needs were identified, and that they were made aware of what help was available to them. Taking a sensitive approach to asking the questions was recognized as an important component of emotional care, preventing people from feeling that they were “not coping” if they were having difficulties, especially when this related to issues of emotional need.

*“I would just talk about whatever [service] it was. Like say, you know, it might be valuable to do this … or it might be valuable for you to do this.”*

(W FG HP03)

*“I think that when you’re trying to integrate a service that’s got stigma attached to it or that people might feel …, don’t want to admit that, you know, they need extra help.”*

(PC041)

Critical to easing the burden and the provision of emotional care was acknowledging people as individuals with unique and dynamic needs.

*“it needs to be individualised. If you’ve got a child with cancer your needs are far different to what having a husband with cancer is, having cancer yourself, it’s just got to be … individualised, tailored.”*

(R FG CC 011)

*“I did ask, I’m really struggling, and maybe the staff could see that. So, you know, having staff at the hospitals that see that … is pretty important.”*

(PC046)

### 3.5. Emotional Care—Everyone’s Business

The provision of emotional care was universally considered to be the responsibility of all health professionals. Having a person’s concerns or needs respected or acknowledged, even if a clinician could not directly deal with them, was recognized as important to addressing a person’s emotional wellbeing.

*“I think that concept of, you know, the word respect and taking the time to get to understand the person you’re speaking with.”*

(M FG HPC07)

*“You want them to have the attitude of this is about you, so we’re gonna answer your questions and if I haven’t got time to answer them now we’re gonna organise a time where we’re gonna sit down and we’re gonna go through all your questions.”*

(M FG PC08)

Communication skills were identified by almost every patient and carer as being essential to the provision of emotional care. Strategies such as making eye-contact, introducing oneself at the beginning of the consultation, using the patient’s name, having read case notes prior to the consultation, and having an un-rushed manner were described as making the difference between feeling emotionally supported and recognized as a person, not just a patient.

*“People who come in … and then they sit there with your notes and proceed to, um, ask you questions, and I have on one occasion had to tell a registrar you should be reading those notes prior to coming in to our consultation otherwise, um, our consultation is not going to be very meaningful …. So that sort of person I wouldn’t talk to about my fear of recurrence for example because I would think they haven’t even bothered to read the notes before walking in so what’s their care factor.”*

(PC049)

Many health professionals spoke of the need to introduce conversations that were about addressing emotional care with a lead-in, prefacing offers of support with an outline of the general concept of care that extends beyond medical treatment.

*“you have to preface it by saying I’m giving you this form to have a look at and complete because we know that we can provide you with extra services but we don’t know specifically what we think you need.”*

(PC040)

Some spoke of time taken to sit with someone in their distress, listen carefully to understand their needs, and ask gently probing questions as being an investment in avoiding difficulties escalating, or even reaching a crisis point before being addressed.

*“I think the biggest thing …is … it’s actually having a conversation with the person…actually sit down and … you might refer them [there].”*

(M FG HP 010)

People affected by cancer and health professionals identified these components of care as being central to provision of emotional support. The ability to ask any health professional about their needs or concerns, and health professionals’ willingness and ability help patients find the most appropriate support either directly or via a referral, were highly valued aspects of emotional care.

*“it’s part of routine supportive care to actually do this identification of need … to do that in a way that’s palatable to patients, and in a way that facilitates …them getting access to the right supports at the right time.”*

(PC041)

However, complex barriers, such as lack of time to address emotional needs or having the skills required to initiate and manage emotional discussion, were acknowledged by all participants.

*“There’s just that everyone is so very busy, so taking time to stop and actually think about what the patient might want or need is hard work … especially ward nurses … they’re under such pressure. And then I think there’s people that don’t have the skills to ask those questions …you know, some people are frightened to ask patients about how they’re feeling properly because they are frightened they won’t be able to deal with that or, you know, be able to help them once they tell them.”*

(PC042)

The tension between demands on health professionals’ time to meet large patient caseloads whilst having only a short amount of time allocated to each patient, was recognized.

*“you don’t want to be a burden … you know people are busy”*

(MFG CC 03)

*“so they [health professionals] would say yeah we’re aware that patients are distressed, we’re aware that it’s a terrible time, but you know, we’re trying to deal with their physical needs, we’re trying to get through the tasks that are required to get them physically well.”*

(P040)

### 3.6. Emotional Care—Anytime, Every Time

The provision of emotional care was recognized as fundamental to wellbeing across all stages of a person’s cancer experience.

*“I think the early stages is where a lot more supportive services should be in whether you want them or not, because the first thing’s shock, grief, you know, who do I talk to, do I tell the kids, no I don’t, do I or don’t I and how do you tell them… I think the end of life there’s a lot more research done into dying and death and there’s all that. It’s that first three months is the hardest.”*

(R FG CP 07)

For some participants the early stages of coming to terms with a diagnosis and engaging with treatment were felt to be so overwhelming that it was difficult to take anything else in and the importance of revisiting needs over time was highly valued.

*“And even during treatment, so I had surgery and then chemotherapy and then radiotherapy, during most of that time I didn’t really actively seek anything because I was so involved in just trying to get through my treatment.”*

(PC046)

*“this notion that … just identify and then refer, it’s short-sighted because things change and new things come up”*

(CP041)

The importance of continually screening for, assessing, and intervening (as appropriate) for emotional needs was emphasized by all patients and carers, emphasizing the dynamic nature of the emotional impost of a cancer diagnosis from diagnosis through to end of treatment and on into follow up care.

*“once you’ve finished your active treatment, going back into the real world, you know, forming your new normal life, what’s normal, because life has changed, and everyone looks at you like oh you’re looking a lot better, your hair’s grown back and you’re looking healthy, so people think everything’s fine but it’s not because things have changed and you’ve got those fears and that sadness still there. So that’s the crucial time where support’s needed.”*

(PC046)

However, irrespective of when support was provided, people affected by cancer were unanimous in their view that dedicated time and space was essential to enable meaningful emotional care, and that attention to emotional needs should be recognized as an intentional, purposeful, and distinct component of cancer care.

*“I think it’s difficult emotionally for patients to hold both sort of stances at once sometimes, to be thinking about the impact of their treatment on their life and what it means to them as a person, at the same time as trying to focus on the side effects of treatment and are those needs being addressed. I think having it separate, there is a role for that, even though we want of course all clinicians to have a certain level of understanding of [emotional care] and represent that in their practices.”*

(CP041)

## 4. Discussion

This qualitative study explored insights from 47 people affected by cancer and health professionals through a series of six focus groups and eight in-depth interviews, that focused on perspectives of emotional care as a component of cancer care. Four key themes were generated focusing on, the centrality of emotional care within the concept of cancer care; the importance of easing the burden through intentional emotional care; emotional care as everyone’s business; and the provision of emotional care as an enabler of emotional wellbeing across the entirety of a person’s cancer experience.

The purpose of this paper was to explore the assumption that the provision of emotional care should be the platform upon which all other aspects of cancer care are delivered (emotional care anytime, every-time) and that without attention to emotional care, no other aspects of cancer care can be fully realized [4]. The paper set out to consider this assumption through perspectives of emotional care as recounted by patients with cancer, carers, and health professionals who had taken part in focus groups and in-depth interviews as part of a larger cancer supportive care study. Findings presented appear to support the assumption, whilst acknowledging that this is an initial exploratory study, representing insights from a predominantly female sample (patients and nurses) from metropolitan settings. As such, further research is needed to test this assumption more fully.

The importance of attending to the emotional stressors or burdens experienced following a cancer diagnosis has long been acknowledged, recognizing that cancer results in atypical levels of fear, worry, distress, and uncertainty for people affected by cancer [30]. Nevertheless, evidence demonstrates that the emotional needs of cancer patients (and those close to them) continue to be overlooked with damaging consequences such as inability to adhere to treatment [3,31,32]. This is not to suggest that cancer health care professionals lack care or empathy, but rather that they face considerable barriers within busy and often under-resourced health care settings to attend to needs beyond the cancer itself or to manage side-effects and symptoms of the treatment or the disease [30,33]. When integrated as the basis upon which cancer care is provided, actively asking about and listening to the needs of patients; empathy for their concerns; going beyond the minimum care provision required; timely identification and response to needs or concerns and, recognizing the needs of family members or carers have been described as powerful and practical ways to mitigate the emotional impost of cancer [30].

Our findings support many of these important insights. Our data demonstrate the importance placed by people affected by cancer and health professionals on the provision of emotional care and the creation of a safe emotional space where concerns and needs can be shared without fear of feeling a burden or as someone unable to cope. Skilled, empathic communication and the delivery of respectful person-centered care were identified as important components and enablers of emotional care and easing the burden. These are not new insights and are recognized as central tenets of patient centered care, benefits of which include enhanced patient experience, improved health outcomes, adherence to treatment, and improved health care costs [34], all of which have been shown to be sensitive outcomes to the provision of emotional care [15,16]. Consistent with other literature, our study demonstrated that the provision of emotional care is central to patients’ experiences and outcomes of their cancer [35]. Our data also captured the importance of ensuring the availability of emotional care across the entirety of a person’s cancer experience. Feeling able to talk to health professionals about emotional concerns, receiving information about services available, having needs acknowledged and validated by health professionals with the skills and knowledge to respond in a humane and respectful way, were critical to seeking and receiving impactful emotional care. These are important insights. With growing numbers of people being diagnosed with and living beyond cancer and its treatments [36,37], there is urgent need to enhance peoples’ capacity to self-care (as they are able) to sustain their wellbeing through provision of timely emotional care. With a global health workforce shortage and reduction in health budgets in real terms [38,39,40], health systems will be unable to sustain the level of care currently provided to people with cancer. Enabling those who can access and proactively use emotional support services to do so will reduce burden on the health system, targeting scarce resources to those with greatest need for specialist emotional support and care. As health professionals there is opportunity for us to look outside acute health services and partner with not for profit, support, and advocacy groups who can deliver front line emotional care to people affected by cancer. Patients and carers in our study did not expect health professionals to have all the answers or resources to hand, indeed they were quick to recognize the limitations and burden on their time and capacity to deliver emotional care, but they did expect that health professionals would access or refer them to services available to meet their needs.

Patient and carer participants in this study noted that the absence of accessible emotional care resulted in feelings of isolation, of fear of missing out on care, and of being a patient in a system, rather than a person enveloped by support. Lack of information about where and how to access emotional support was described as exacerbating already complex situations, compounding feelings of isolation and fear, and provision of support without taking the time to understand a person’s unique and dynamic needs potentially negated the opportunity for receipt of meaningful emotional care—impacting not only emotional wellbeing but cancer outcomes. Recently published evidence has demonstrated considerable return on investment for patients and health systems when patients receiving timely supportive care, inclusive of emotional care [2]. Our data support these findings, demonstrating advocacy for timely and ongoing access to emotional care for patients and carers, and investment in health professional knowledge and capability to deliver emotional care to prevent and mitigate the emotional impacts of a cancer diagnosis. Importantly, our data demonstrate that emotional care is a critical domain in the provision of cancer supportive care, requiring effective communication skill and empathy on the part of health professionals, and investment in time and resources by health services to enable and health professionals to deliver the care they recognize and value as components of patient-centered care.

An important insight from our study is that the provision of emotional care requires distinct and purposeful attention recognizing that it has to compete with the urgency of treating the cancer itself, “*…it’s difficult emotionally for patients to hold both sort of stances at once sometimes, to be thinking about the impact of their treatment on their life and what it means to them as a person*”. Participants in this study demonstrated the importance of creating an emotionally supportive context for the provision of the totality of cancer care, enabled through respectful interaction, provision of time and attention to individual needs, skilled communication and readiness to source and refer patients and carers to a diverse range of support services and resources.

The strengths of this study are that it generated data from a cohort of patients, carers, and health professionals with lived experience of providing and receiving cancer supportive care. The intent was not to generate generalizable data but to explore in-depth perspectives on emotional care as a component of cancer care. Data included participants living and working across metropolitan and regional areas of one state in Victoria, Australia. The paper provides novel insight on emotional care, offering insights to ways in which the experience of emotional care can be enhanced for consumers and enabled by health professionals. Future studies may focus on the impact of interventions to strengthen provision of emotional care, patient reports of care experiences, health outcomes, and system impacts. Insights from the focus groups and interviews were fed back to participants as part of the larger study. A key limitation of the study is that no people from Aboriginal or Torres Strait Islander or culturally or linguistically diverse peoples were included. As such, these findings are likely to present “the best’ of experiences, and recommendations generated may have limited relevance to under-served populations.

## 5. Conclusions

In conclusion, emotional care is critical to the provision of patient-centered care, without which effective delivery of other aspects of cancer care may be hindered. The provision of emotional care and perceptions of its adequacy or impact rely on health professionals’ communication skills; their ability to offer proactive opportunity for discussion of needs and concerns across all stages of the cancer experience, and importantly, their access to time and resources necessary to elicit and respond to emotional needs. For patients, availability of opportunity, encouragement to voice, and awareness of the legitimacy of emotional concerns are important facilitators of emotional care. Future studies are needed to test interventions to enhance provision of intentional and purposeful emotional care delivery to ensure patients achieve the best health outcomes possible. Studies focusing on the emotional care needs of underserved populations are urgently needed.

## Figures and Tables

**Table 1:** Examples of focus group/interview questions.

Exploratory Questions
What does the term supportive care mean to you?
Can you think of a time you have had a positive “supportive care” experience?
What skills and experience do you think clinicians working with people affected by cancer need to have?
What do you think is most important or valuable about supportive care?

**Table 2:** Participant demographics (n = 47).

Characteristics	Patients and Carers (n = 22)
Female	21 (95%)
Age (median and range)	63 (44–86)
Patient	16 (72%)
Family member/carer	6 (28%)
*Place of residence*	
Metropolitan	14 (64%)
Regional	7 (32%)
Not specified	1 (5%)
*Language spoken at home*	
English	100%
**Characteristics**	**Health care participants (n = 25)**
Male	3 (11%)
Female	22 (89%)
Age (median and range)	49 (27–63)
*Profession*	
Nurse	10 (36%)
Dietician	4 (14%)
Medical oncologist	3 (10%)
Psychologist	2 (7%)
Social Worker	1 (4%)
Physiotherapist	1 (4%)
Exercise physiologist	1 (4%)
Counsellor	1 (4%)
Radiation Therapist	1 (4%)
Clinical Trials Coordinator	1 (4%)
*Place of work*	
Metropolitan	15 (60%)
Regional	8 (29%)
Rural	2 (7%)

## Data Availability

The data are stored electronically and in accordance with Good Clinical Practice requirements in the Academic Nursing Unit, The Peter MacCallum Cancer Centre, Melbourne Australia, De-identified data can be accessed from the lead author (MK).

## Supplementary Materials

The following supporting information can be downloaded at: https://www.mdpi.com/article/10.3390/healthcare11040452/s1, File S1: People affected by cancer: Focus group/interview schedule.
