# Supplementary material for: Perspectives on Emotional Care: A Qualitative Study with Cancer Patients, Carers, and Health Professionals
Source: Healthcare (Basel). 2023 Feb 4;11(4):452. doi: 10.3390/healthcare11040452 (PMC9956222; doi:10.3390/healthcare11040452)
Supplement: Supplementary file 1 [file healthcare-11-00452-s001.zip › File S1.pdf]

People affected by cancer: Focus group/interview schedule

Q1) We are interested to learn about peoples' experiences when first diagnosed with cancer and the impact it has had on you and your families' lives

Focus Group/Interviewee notes: looking for responses around importance of supportive care and emotional care specifically to patients and families,

Q2) Supportive care can many different things to different people. What does supportive care mean to you?

Focus Group/Interviewee notes: Elicit responses around meaning and importance of supportive care (as feeling cared about- emotional care), that supportive care should be personalised to each individual.

Q3) Once conversations and decision about treatment had been made, did anyone talk to you about support services or resources available to you, that would have made your situation easier?

Focus Group/Interviewee notes: Can provision of supportive care ease the burden for peopel in their everyday lives, if so, how? What matters and why? Exploring emotional burden.

Explore and probe: who spoke about supportive care; at what time, and what kind of help did they offer? How did it make you feel when you were asked about your supportive care needs?

Focus Group/Interviewee notes: Explore importance of timing, need for clinicians to consistently check in over time/ across journey. Elicit further response around importance and value of Supportive Care (feel), did you feel listened to, cared about (emotional care) If yes, how, what was important and why?

Probe: If no one spoke about supportive care, looking back, would it have been helpful to have had someone ask you if you wanted/needed any help or support?

Q4) If you didn't feel you needed help or support, was there a member of your family or support network who would have benefitted from help?

Focus Group/Interviewee notes: Value and importance of Supportive Care not only just for the patient, how itcan ease the burden for family members (Supportive Care for everyone-attending to the emotional needs of family members)

Q5) The term supportive care is used to describe a wide range of help, services, referrals available to people affected by cancer. Did anyone talk to you about what was available to support you?

Focus Group/Interviewee notes: Supportive Care involves providing the right information (needs based) at the right time. SC needs to be offered proactively, and across multiple domain?

Q6) In what context or way were you told about supportive care?

Focus Group/Interview notes: Explore communication skills required to provide effective supportive care, as a part of usual care and to convey emotional care.

Q7) Is it important that people are made aware of the kinds of support available to them? If yes, why do you think this is so important?

Focus Group/Interviewee notes: Explore value and importance of Supportive Care being offered, ease of burden (including emotional impost) on patient/family. Explore expectations of clinicians – e.g., they don't have to provide the Supportive Care, but give the patient the information/referral pathway for them to access the SC required?

Q8) Who do you think should ask patients/ their families whether they want/need help or support? When do you think people should be asked?

Focus Group/Interviewee notes: Explore perceptions of clinicians in the conversation about Supportive Care and the need to get the timing right and to do so over time.

Q9) Can you tell me about supports or help you have received or found for yourself and whether these were helpful for you? If so, in what way?

Focus Group/Interviewee notes: Explore views regarding whether Supportive Care needs to be proactively offered and its importance/value. Is emotional care overt in responses?

Q10) If you could give a person who has just been told they have cancer one message about support services, help or referrals – what would that be?

Focus Group/Interviewee notes: Explore why Supportive Care is important, why health professionals should ask about what services are available

Q11) What has been the most helpful advice, resources, support or referrals you have received, and how did they make a difference to you

Focus Group/Interviewee notes: Explore benefit of early Supportive Care intervention as able to reduce burden; ability to access it when/how it suited them….personalised care.

Q12) Thinking back over your experience, can you identify a resource or a support that would have been helpful but wasn't offered or talked about with you? If so, can you tell me about it?

Focus Group/Interviewee notes: Explore what was needed and the difference it could have made – do people talk about emotional needs?

Q13) Did you seek or get support from outside of the hospital system, for example your GP, local council or the Cancer Council?

Focus Group/Interviewee notes: To what extent do people know what's available to them?

Q14) Why is it important for health care professionals to ask about supportive care needs?

Focus Group/Interviewee notes: Explore why health professionals should ask about Supportive Care needs; what is the importance/value in having health professionals ask about Supportive Care (emotional needs), and tailoring it to individual circumstances.

Health Care Professional" Focus group/interview schedule

Q1) We are interested to learn about health care professionals' experiences of cancer supportive care

Focus Group/Interviewee notes: looking for responses around importance of supportive care and emotional care specifically to patients and families

Q2) Supportive care can many different things to different people. What does supportive care mean to you?

Focus Group/Interviewee notes: Elicit responses around meaning and importance of supportive care (as feeling cared about- emotional care), that supportive care should be personalised to each individual.

Q3) Once conversations and decision about treatment had been made, did you talk about support services or resources/point patients and their family members to resources available?

Focus Group/Interviewee notes: Explore knowledge and awareness of services. Explore importance of timing, and need for clinicians to check in over time/ across journey as needs change.

Q4) Do you talk to patients/carers about supportive care or does another member of the team do this? If yes, in what context or way do you talk about supportive care?

Focus Group/Interview notes: Explore perceptions about "who's business supportive care is" Doe different health professionals address different aspects, e.g., emotional care?

Q5) Is it important that people are made aware of the kinds of support available to them? If yes, why do you think this is so important?

Who do you think should ask patients/ their families whether they want/need help or support? When do you think people should be asked?

Focus Group/Interviewee notes: Explore value and importance of Supportive Care being offered, ease of burden (including emotional impost) on patient/family. Explore expectations of knowing about and being able to refer patients to Supportive Care services/resources as a clinician.

Q6) Can you tell me about supports or help you recommend and why?

Focus Group/Interviewee notes: Explore views regarding whether Supportive Care needs to be proactively offered and its importance/value. Is emotional care overt in responses?

Q7) If you could give a person who has just been told they have cancer one message about support services, help or referrals – what would that be?

Focus Group/Interviewee notes: Explore why Supportive Care is important, why health professionals should refer patients to services that are available

Q8) What do patients tell you have been the most helpful advice, resources, support or referrals you have directed them to, or you have provided yourself, and why?

Focus Group/Interviewee notes: Explore benefit of Supportive Care interventions as able to reduce burden and in what way?

Q9) Thinking back over your experience, what do you think are the most overlook and the most important supportive care resources/services/interventions and why?

Focus Group/Interviewee notes: Explore common needs and whether emotional needs are identified as common?

Q10) Did you refer patients or turn for advice for Supportive Care from outside of the hospital system, for example the Cancer Council?

Focus Group/Interviewee notes: To what extent do health professionals know what's available to them? To what extent do health professionals see provision of Supportive Care as the business of acute health settings?

Q11) Do you believe it is important for health care professionals to ask about supportive care needs?

Focus Group/Interviewee notes: Explore why health professionals should ask about Supportive Care needs; what is the importance/value in having health professionals ask about Supportive Care (emotional needs) and tailoring it to individual circumstances.
